# Reproduction and Feeding of the Electric Fish *Brachyhypopomus gauderio* (Gymnotiformes: Hypopomidae) and the Discussion of a Life History Pattern for Gymnotiforms from High Latitudes

**DOI:** 10.1371/journal.pone.0106515

**Published:** 2014-09-10

**Authors:** Julia Giora, Hellen M. Tarasconi, Clarice B. Fialho

**Affiliations:** Laboratório de Ictiologia, Departamento de Zoologia, Universidade Federal do Rio Grande do Sul, Porto Alegre, Rio Grande do Sul, Brazil; Universität Bielefeld, Germany

## Abstract

The reproductive biology and feeding habits of the electric fish *Brachyhypopomus gauderio* were studied. The species has seasonal reproductive behavior, with breeding occurring during the Southern Hemisphere spring and summer, and having a positive relation with the photoperiod variation. *Brachyhypopomus gauderio* was defined as a fractional spawner, with low relative fecundity and high first maturation size. Sexual dimorphism was registered, males undergoing hypertrophy of the distal portion of caudal filament. The results on reproductive biology herein obtained are in agreement with data concerning gymnotiforms from Southern Brazil and Uruguay, pointing to an ecological pattern for the species from high latitudes, differing from species with tropical distribution. According to the analysis of the food items, *B*. *gauderio* feed mainly on autochthonous insects, likewise the other gymnotiforms previously investigated, leading to conclude that there is no variation on the diet of the species of the order related to climatic conditions or even to habitat of occurrence.

## Introduction

Gymnotiformes have a wide geographical range throughout South and Central America, occurring in an incredible diversity of aquatic habitats including flood-plains, flooded forests, forest streams, cataracts, swamps, coastal creeks, estuarine reaches, and mainly in river channels [1]. Because of the geographical wide distribution of the order, its integrants are exposed to tropical weather environments, presenting drastic changes in water level and niche availability, as well as to temperate weather environments, presenting great seasonal changes in photoperiod and temperature. The adaptative response of the gymnotiform species to these different weather scenarios has been reported in many studies [2]–[19].

The genus *Brachyhypopomus* is included in the Hypopomidae family, which is distributed from Río de La Plata in Argentina (35°S) to northern Panama (8°N) [20]. The genus prefers slow-moving and shallow waters with dense floating vegetation that can be used as shelter and sites with leaf mats in the bottom [21]. The Amazon-Orinoco-Guianas superbasin is the center of diversity for the Gymnotiformes [22]; in the same way, *Brachyhypopomus* species reach the highest diversity and abundance in Amazon flood-plains [23]. According to a continuously updated catalog of fishes [24], the family Hypopomidae presents 25 valid species, 10 of each described in the last 10 years. Of these 10 species recently described, 4 are *Brachyhypopomus* species from southern and southeastern Brazil and Uruguay [25]–[28], showing that this regions were poorly studied and are more taxonomic diverse than it was previously previewed.

*Brachyhypopomus gauderio* was recently described [28] as a species formerly identified as *B*. *pinnicaudatus*. The species is widely distributed from the Laguna dos Patos, Rio Uruguay and Rio Tramandaí drainages in the Rio Grande do Sul state, Brazil, also occurring in the Rio Uruguay basin in Uruguay and in the Rio Paraguay basin in Paraguay [28]. The species is abundant and easily found from central, southern, and coastal regions of the Rio Grande do Sul state, and it is commonly syntopic with other *Brachyhypopomus* species, especially *B*. *draco*.

Recently, studies concerning the ecology and life history of species of the genus *Brachyhypopomus*, occurring in Rio Grande do Sul state and Uruguay, has improved the knowledge about this fishes inhabiting the southern boundary of the genus distribution, and has made this genus the most understood group of Gymnotiformes in terms of life history aspects [17], [19].

Despite all increasing knowledge on reproduction and feeding of gymnotiforms from southern Brazil, and more specifically on species of the genus *Brachyhypopomus*, there is no information about life history strategies of *B*. *gauderio*. Therefore, this study aims to investigate the aspects of reproductive biology and feeding habits of *B*. *gauderio*, as well as to compare these results with the existent information for gymnotiform species from the southern boundary of the order distribution in order to establish life history patterns.

## Materials and Methods

### Study area

**Figure 1:**
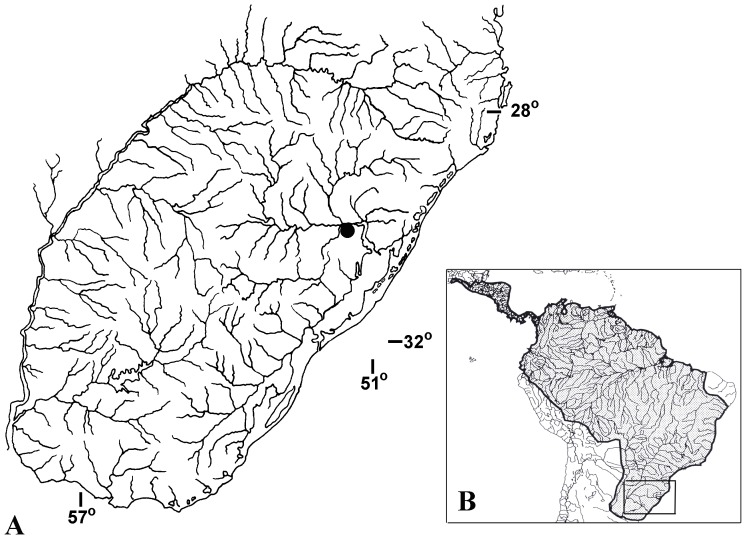
Sampling site of *Brachyhypopomus gauderio*. Geographic location of the sampling site of *Brachyhypopomus gauderio*. A: sampling site of *B*. *gauderio* in the Rio Grande do Sul State, Brasil; B: distribution of the order Gymnotiformes through the Neotropical region.

*Brachyhypopomus gauderio* specimens were sampled in a flooded area near Arroio dos Ratos creek (29°57′31,9″S 51°33′10,1″W) in Charqueadas municipality, which integrate the Laguna dos Patos basin in Rio Grande do Sul, Brazil (Fig. 1). The sampling area presents dark and slow-moving water, with hardly any flow, muddy bottom and depth ranging from 1 to 1.5 meters, with very little depth variation among the months of the year. The location has also abundant floating vegetation, manly composed by *Pistia stratiotis* and *Salvinia auriculata*. Marginal rooted vegetation is mostly composed by *Polygonum* sp., reeds and bushes. In addition to *B*. *gauderio*, the gymnotiforms *Brachyhypopomus draco*, *Eigenmannia trilineata* and *Gymnotus* aff. *carapo* were sampled at this locality.

### Sampling

Field work and sampling were executed according to the Authorization for Scientific Activities (number 873510) concede by the Instituto Brasileiro do Meio Ambiente e dos Recursos Naturais Renováveis - IBAMA. The IBAMA is the Brazilian Government Department responsible for analyzing research projects that involve field work, capture and sampling of specimens of native fauna and flora, conceding authorizations which regulate the area and period of sampling, as well as the species and number of specimens which can be capture and/or sacrificed during a study. IBAMA Authorization for Scientific Activities is obligatory for any research project that includes field work in Brazilian territory.

Ethical approval was not obtained because it was only required for animal experimental studies and/or studies concerning capture and/or sampling of Tetrapoda according to Brazilian scientific ethical committees. Brazilian scientific ethical committee concerning animals other than Tetrapoda was only created in 2009, after the field work related to this manuscript. The collections were undertaken monthly from April/2005 to March/2006 between 10:00 am and 16:00 pm under floating vegetation using a dip net and an electric fish finder [29]. Aiming to avoid animal suffering, the specimens were euthanized by emersion in eugenol 10% solution in ethanol 99%, and fixed just after in 10% formalin solution. Water temperature, conductivity, pH and dissolved O_2_ were recorded at the time of sampling. Rainfall data were obtained from the Meteorology District of Porto Alegre.

In the laboratory, fish were transferred to 70% ethanol. After approximately one week in the ethanol solution, total length (Lt) in millimeters and total weight (Wt) in grams were measured and individuals were dissected to record intestine length (Li) in millimeters, and stomach (Ws) and gonad (Wg) weight in grams. Due to the fact that organ weights were always analyzed as percentages of fish total weight, dehydration after transference from formalin to ethanol was not considered. Voucher specimens were catalogued in the fish collection of the Departamento de Zoologia, Universidade Federal do Rio Grande do Sul, Porto Alegre, Brazil (UFRGS 9200).

### Data analysis

Stomach repletion index (RI) and gonadosomatic index (GSI) were estimated following the formula adapted from Santos [30]. These indexes represent the percentage organ weight related to fish total weight: RI  =  Ws x100/Wt and GSI  = Wg x100/Wt. Ws corresponds to stomach weight, Wg to gonad weight, and Wt to total weight. The analysis of variance (ANOVA) with Tuckey's post-test was applied to verify significant differences between the monthly GSI values of males and females separately, as well as for monthly RI values of males and females separately. The intestinal quotient (IQ) represents the ratio of the intestine length related to the fish total length: IQ  =  Li/Lt. Li corresponds to the intestine length and Lt to the total length.

The reproductive period for males and females was established through the analyses of monthly variation of the mean GSI values. The multiple regression with analysis of variance (ANOVA) was applied to verify possible dependence between abiotic factors (rainfall, photoperiod, temperature, conductivity, pH, and dissolved O2) and the reproductive period (GSI), and between the feeding activity and the reproductive period [31].

The absolute fecundity was estimated counting all vitellogenic oocytes present in the ovaries of females with the highest GSI values. Nine females were selected for the fecundity analysis. The relative fecundity was determined by the number of counted oocytes per female milligram of weight [32]. For the determination of the spawning type, the same gonads selected for fecundity analysis were used. A sub-sample of 150 oocytes was removed from each selected gonad and the largest possible oocyte diameter was obtained with observation on a stereomicroscope [33].

The sex ratio was determined by the distribution of male and female frequencies during the sampling period. A chi-square test (α<0.05) was applied to verify the existence of significant differences between the number of males and females of *B*. *gauderio*. The size of first gonad maturation of males and females was estimated from the distribution of juvenile and adult relative frequencies for total length classes [33]. The obtained curve was adjusted according to the expression: F  =  1-(e - aLt^b^); F corresponds to the relative frequency of adults, e to the natural logarithm base, Lt to total length (mm), and a and b to estimated constants related to curve adjustment. The first gonad maturation size is considered as corresponding to a frequency of 0.5 (50%) of the adult individuals.

For the determination of the period of breeding of new individuals, the months when larvae were sampled were recorded. The distribution of relative frequencies of males and females in different total length classes was analyzed and tested with a chi-square test (α = 0.05) to observe possible sexual dimorphism related to specimen lengths. Relative frequency of *B*. *gauderio* specimens with vertically broadened and laterally compressed distal portion of the caudal filament was calculated to observe the development of this sexually dimorphic feature along the reproductive period.

Stomach content analysis was performed with the help of a stereomicroscope and the organisms in the stomach were identified to the highest taxonomic level possible. The alimentary items were analyzed by the frequency of occurrence method [34] and by percent composition method [35], where the number of times that each item has occurred was treated as the percent of total occurrence number of all items. For this, the items were grouped in broader taxonomic and ecological categories: microcrustacea (Microcr), digested organic material (DOM), autochthonous insects (AuI), allochthonous insects (AlI), arachnid (Arac), vegetal material (VM), sediment (Sed), and others (Others).

The importance of each of these alimentary categories in the diet of the species was estimated by a semi-quantitative abundance scale, where each category contribution is estimated according to the area that it occupies in relation to total content. The scale used was based on Granado-Lorencio and Garcia-Novo [36]: 0 – absent; 1 – scarce (less than 25%); 2 – frequent (25% to less than 50%); 3 – very frequent (50% to less than 75%); 4 – abundant (75% to 100%). From this scale the index of alimentary importance (IAI) was calculated from the formula [36]: IAI  =  Σ[(XK .k)-1(n-1)]; XK is the frequency of occurrence of a certain diet component Xi with category k; K is the abundance category (0, 1, 2, 3 and 4), and n is the number of categories of the scale. According to Guillen and Granado [37], the main food category is that which presents IAI values above 0.3; the additional food category presents IAI values from 0.3 to 0.15, and the accidental food category presents values below 0.15.

## Results

Overall 211 specimens of *Brachyhypopomus gauderio* were sampled: 108 males with total length ranging from 42.96 mm to 188.8 mm, 102 females ranging from 45.65 mm to 175.36 mm, and 1 larva with 24.23 mm of total length.

### Reproduction

**Figure 2:**
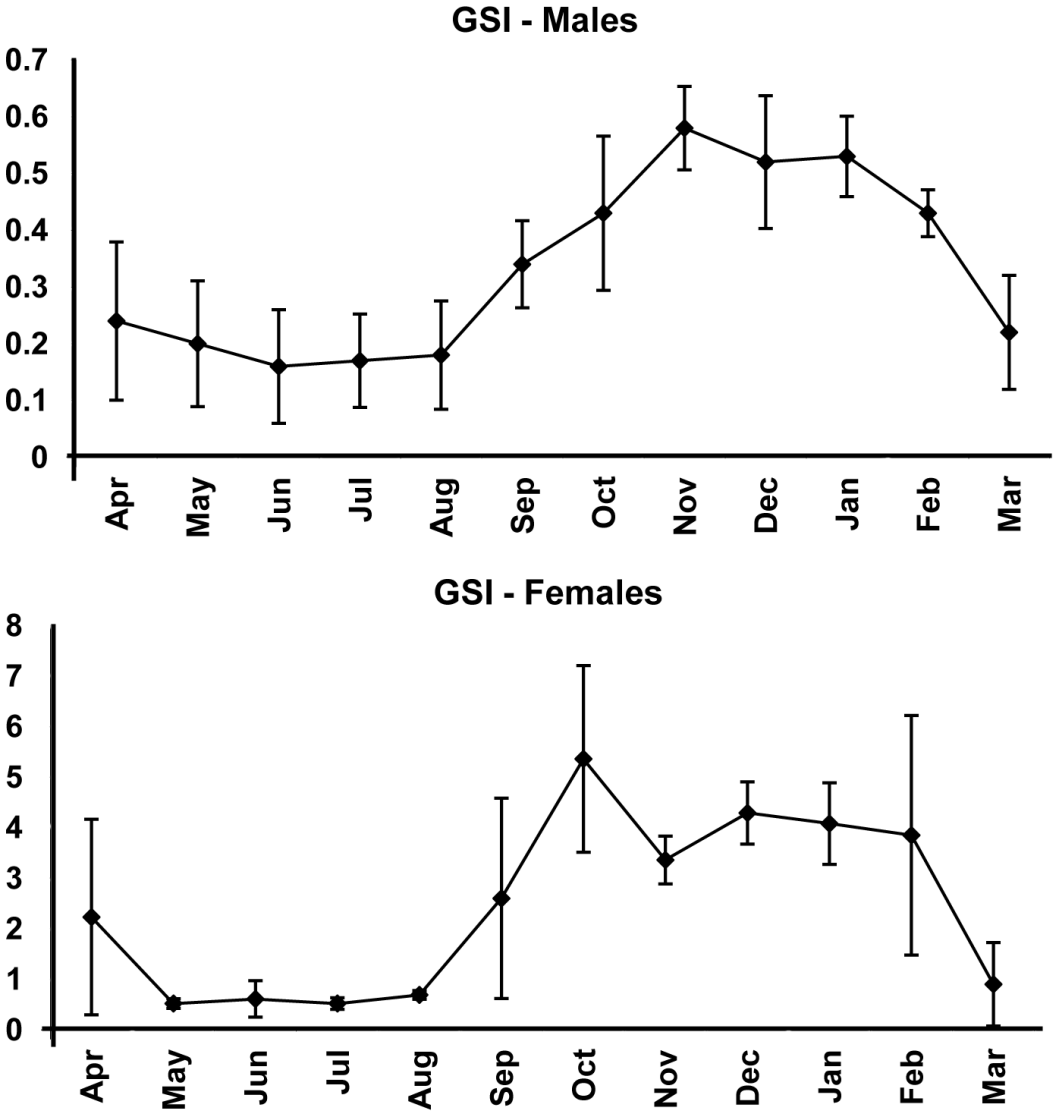
Gonadossomatic index. Monthly variation of mean gonadosomatic index (GSI) for *Brachyhypopomus gauderio* males and females from April/2005 to March/2006. Vertical bars represent the standard deviation. Numbers above the bars correspond to the numbers of specimens included in the analysis.

The reproductive period estimated for *B*. *gauderio* lasted from October/2005 to February/2006, with male GSI peak occurring in November/2005 and female GSI peak occurring in October/2005 (Fig. 2). According to the analysis of variance (ANOVA) with Tuckey's post-test, male and female GSI values differ significantly between the months of sampling (F = 15.26, p<0.05 for males; F = 16.65, p<0.05 for females), the period from October/2005 to January/2006 differing from April to September/2005 and from February to March/2006 in males, and the period from October/2005 to February/2006 differing from April to September/2005 and from March/2006 in females. Parental care behavior was registered for the species given the detection of larval agglomeration under the vegetation along with an adult male during the months of December/2005, February/2006, and March/2006.

The GSI of males and females was not significantly related to the repletion index (RI) according to the test of multiple regression with analysis of variance (ANOVA). Among all the abiotic factors tested, the GSI of males and females presented significant relation only to photoperiod variation (males: F = 114.970, t = 10.722, p = 0.000; females: F = 27.006, t = 5.197, p = 0.000). Monthly data of water temperature, pH, conductivity, dissolved oxygen, rainfall, and photoperiod are summarized in Table 1.

**Table 1:** Abiotic factors at sampling site.

	Temperature	pH	Conductivity	Diss. Oxygen	Rainfall	Photoperiod
Apr					145.8	700
May	19.2	7.22	13.53	0.95	153.7	650
Jun	18.7	7.08	13.34	0.89	34.7	615
Jul	15.3	7.16	13.02	0.89	57.7	619
Aug	17	7.08	12.08	0.9	155.9	668
Sep	18.5	7.22	10.01	1	164.3	727
Oct	20.9	7.17	8	0.8	271.1	784
Nov	24.8	7.25	48.6	1.7	79.3	833
Dec	29.7	7.32	52.7	0.6	56	845
Jan	26	7.29	57.2	0.44	174.2	818
Feb	26	7.29	55.8	0.25	88.9	775
Mar	27	7.27	13.7	0.7	81.2	720

Monthly variation of the water temperature (°C), pH, conductivity (µS/cm), dissolved oxygen (mg/l), rainfall (mm), and photoperiod (min) values in the Arroio dos Ratos creek, Charqueadas municipality, from the period of April/2005 to March/2006. Empty spaces  =  months when no data was registered.

**Figure 3:**
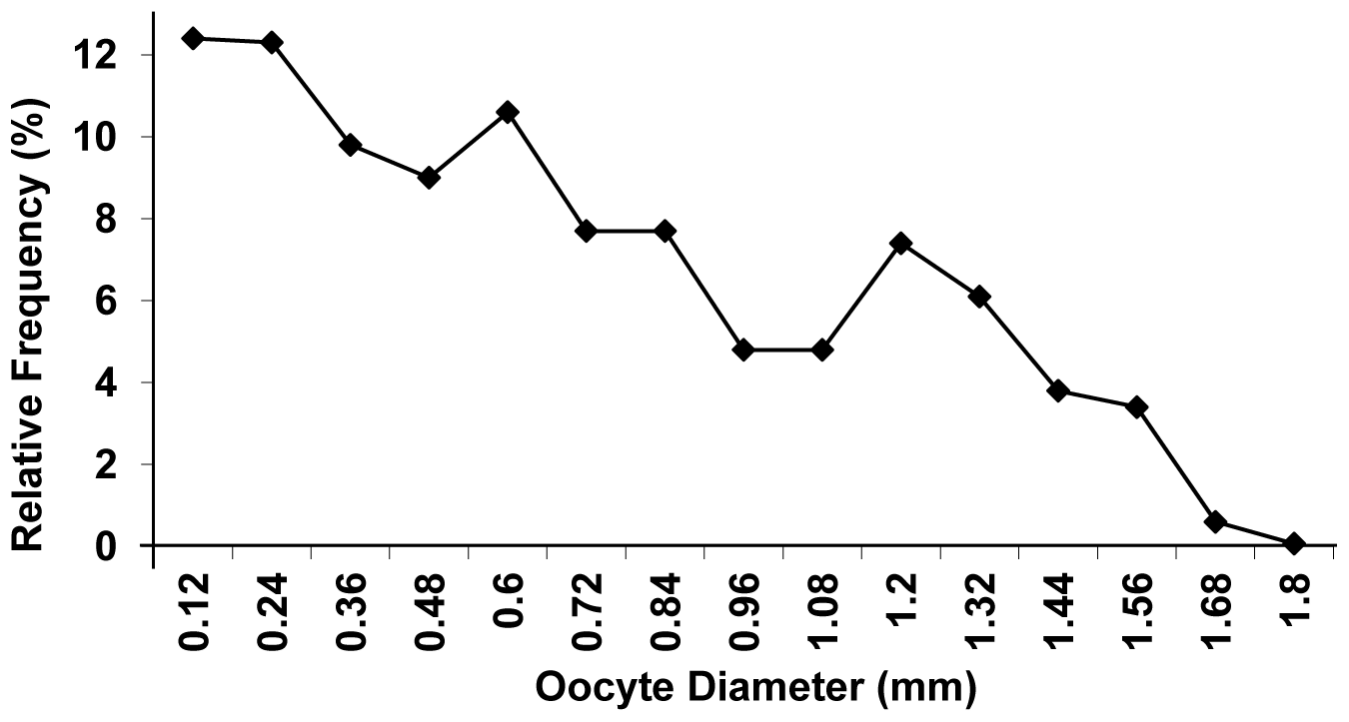
Spawning type. Distribution of the relative frequency of oocyte diameters of *Brachyhypopomus gauderio* mature females. Number of specimens analyzed =  09.

The absolute fecundity had an average value of 589.44 oocytes (ranging from 299 to 799 oocytes) for females with total length from 85.42 a 149.0 mm for (Table 2). The average relative fecundity was estimated as 0.20 oocytes per mg total weight (Table 2). The analysis of the absolute frequency distribution of oocyte diameter corresponds to that of a species with oocyte development synchronic in more than two groups, iteroparity, and fractional spawning (Fig. 3). It is observed a high frequency of store oocytes, which are the smallest oocytes observed in the gonads, have not initiated the vitellogenic process, and will only mature in the next reproductive period. Besides the store oocytes, it is observed in the analyzed mature gonads oocyte shares in successive maturation stages that are eliminated at different times in the reproductive period of the species.

**Table 2:** Fecundity data.

	TL (mm)	TW (g)	GSI	AF	RF
	85.42	1.12	6.29	299	0.27
	112.72	1.57	6.62	466	0.29
	131.05	2.90	6.40	540	0.19
	133.08	3.19	5.84	574	0.18
	133.15	4.23	5.01	734	0.17
	135.13	3.86	4.65	616	0.16
	135.59	3.79	8.93	637	0.17
	145.57	4.65	5.84	799	0.17
	149.1	4.10	5.50	640	0.16
Mean	128.98	3.27	6.12	589.44	0.20

Total length (TL), total weight (TW), gonadosomatic index (GSI), absolute fecundity (AF), and relative fecundity (RF) of nine females of *Brachyhypopomus gauderio.*

**Figure 4:**
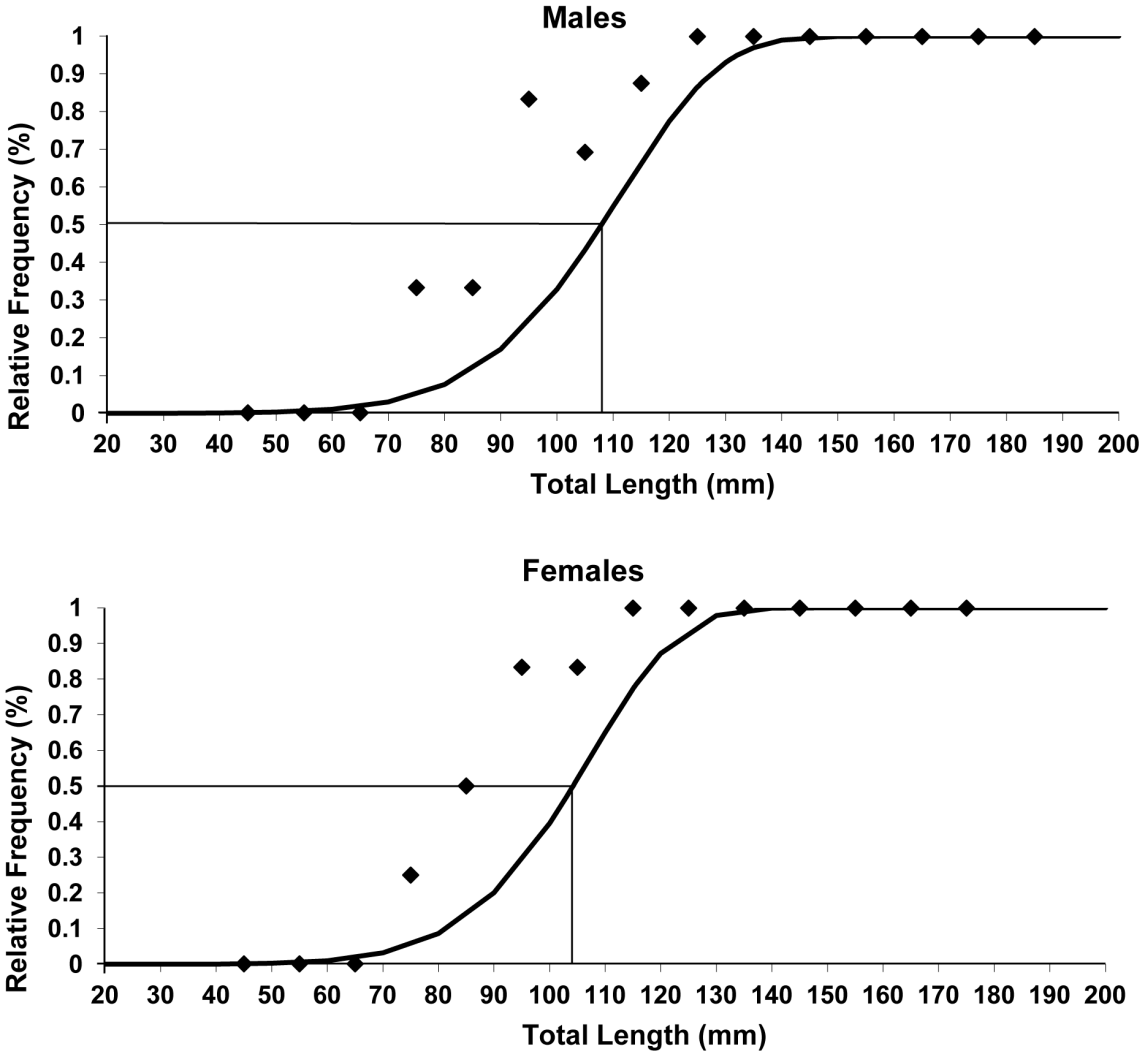
First gonad maturation size. First gonad maturation size of *Brachyhypopomus gauderio*. Distribution of the relative frequency of specimens in total length classes. Lines represent the point at which 50% of the individuals are considered adults. Number of males analyzed =  99. Number of females analyzed =  96.

The first gonadal maturation size was estimated for *B*. *gauderio* males as 108.0 mm and for females as 104.5 mm (Fig. 4). The chi-square test results demonstrate a sex ratio of 1:1 in analyses of the number of males and females monthly sampled, as well as in analyses of the total number of males and females sampled (χ^2^_calculated_ = 0.09; χ^2^_0.05_;1 = 3.84). The period of new individuals breeding was established as occurring from December/2005 to March/2006, these being the months larvae (February/2006), and male and female from the smallest length classes were collected.

**Figure 5:**
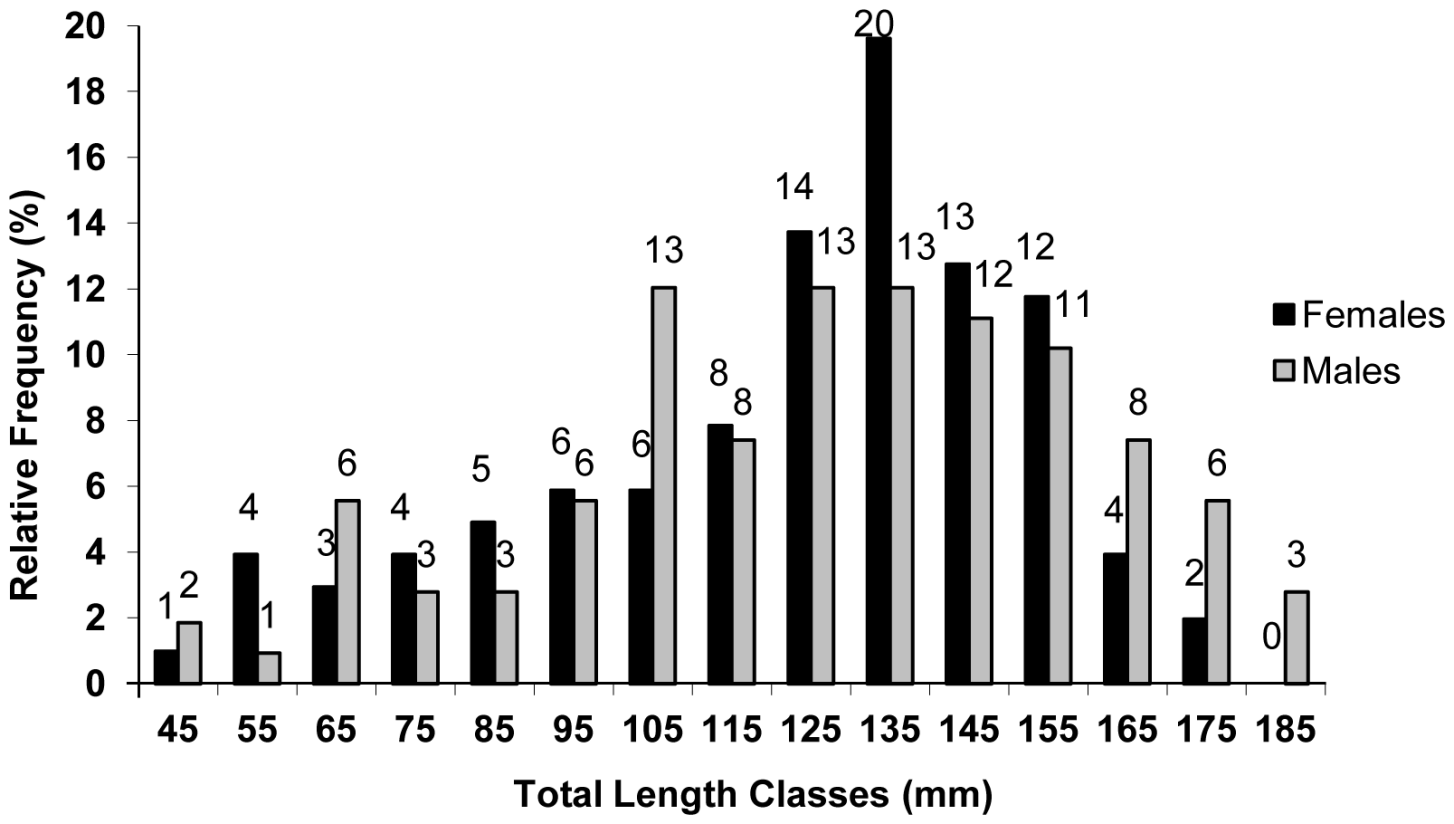
Total length classes. Distribution of the relative frequency of males and females of *Brachyhypopomus gauderio* assigned to total length classes (10 mm total length). Black columns represent females, and grey columns represent males. Numbers above the columns correspond to the absolute values.

**Figure 6:**
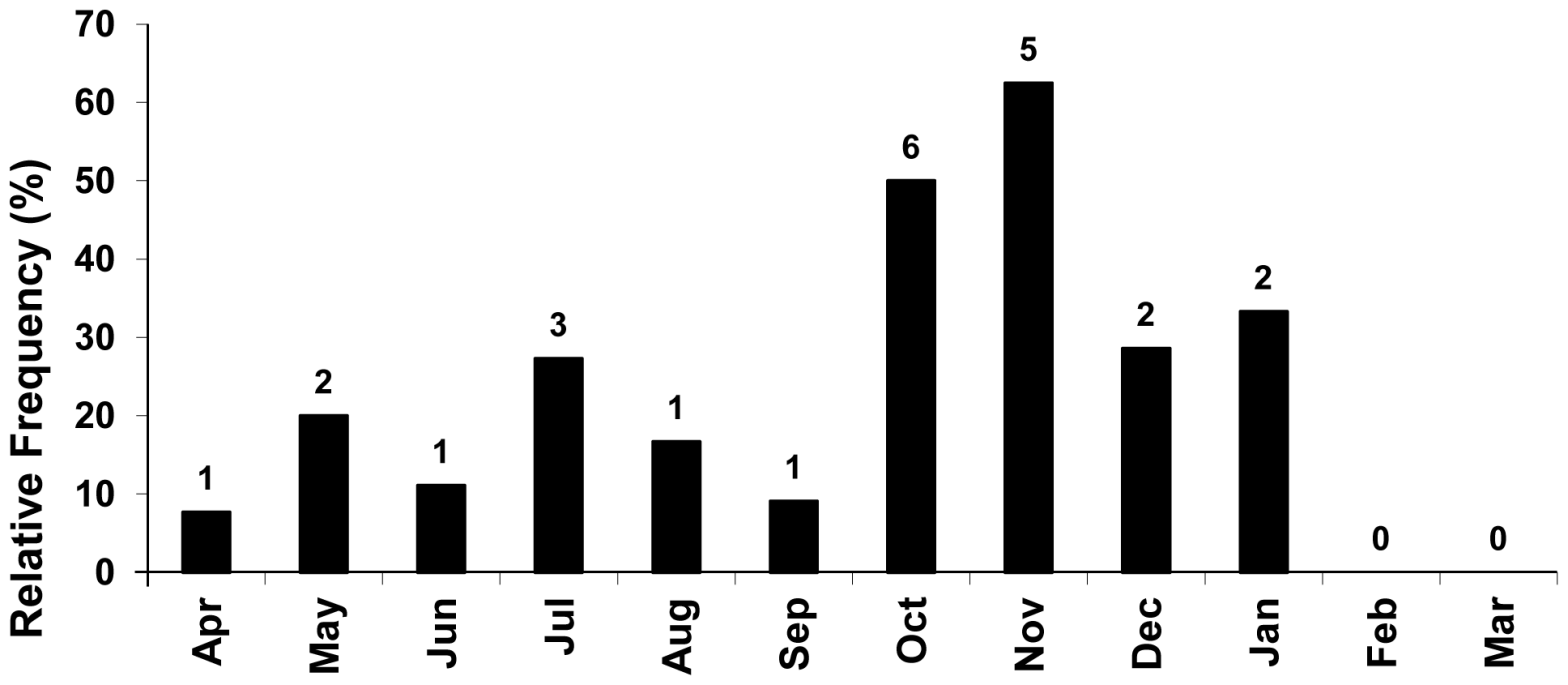
Hypertrophy of male caudal filament. Monthly variation of the relative frequency of males of *Brachyhypopomus gauderio* with hypertrophy of the distal portion of caudal filament. Numbers above the columns correspond to the absolute values.

Significant differences in total length related to sexual dimorphism were not observed for *B*. *gauderio* according to the chi-square test (α<0.05) applied to the distribution of males and females in different total length classes; however, males reached the largest length class established (Fig. 5). Males with hypertrophy of the distal portion of caudal filament – vertical broadening and lateral compression give the distal portion of caudal filament a feather-like shape – were sampled during all year months except February and March/2006 when no males larger than 130 mm were collected. The highest relative frequencies of males with broadened caudal filament occurred in October and November/2005 (Fig. 6). Total length of specimens with caudal filament hypertrophy ranged from 145.38 mm to 188.8 mm, in spite of it, males with total length included in the quoted range could be found without this structure hypertrophied during the same months of the first ones. No females were observed with this caudal filament modification.

### Feeding

**Figure 7:**
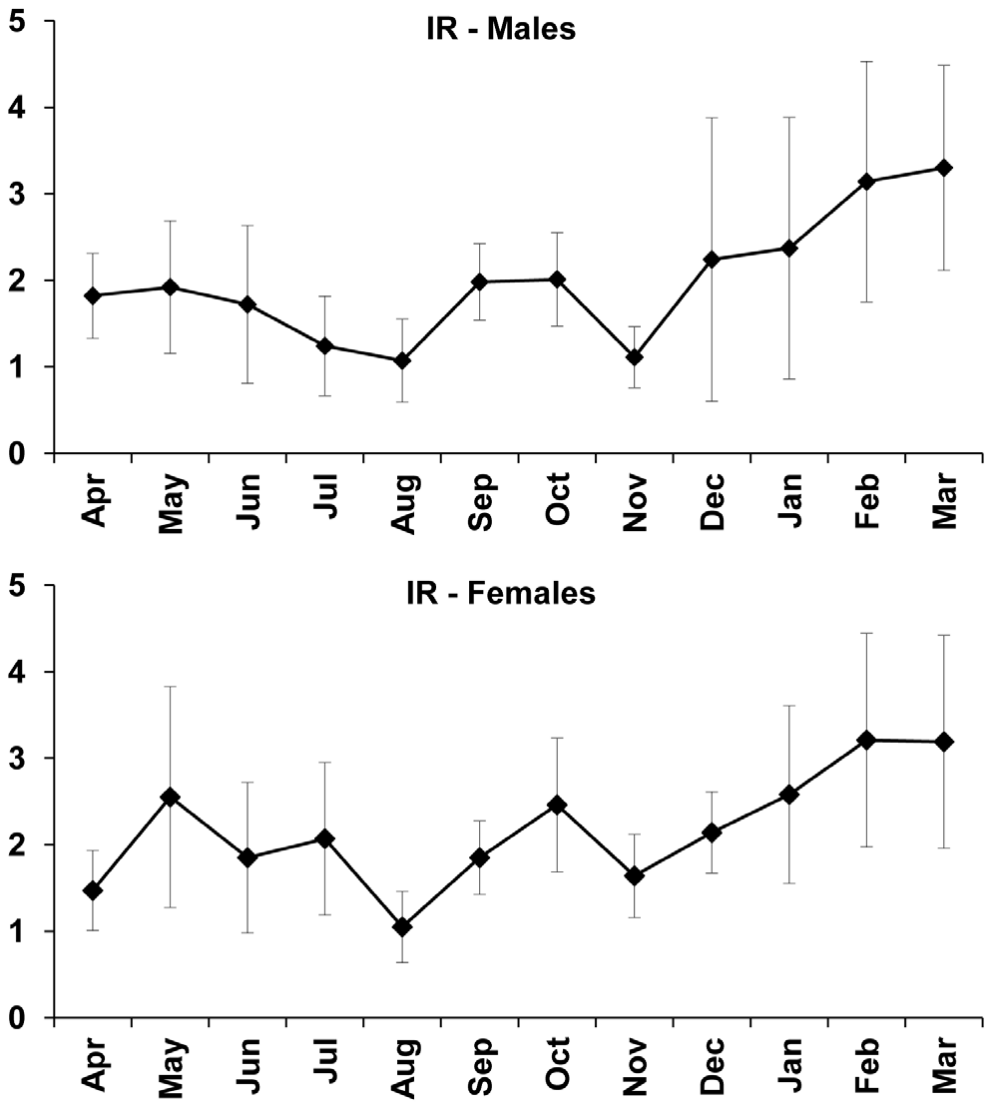
Repletion index. Monthly variation of mean repletion index (RI) for *Brachyhypopomus gauderio* males and females from April/2005 to March/2006. Vertical bars represent the standard deviation. Numbers above the bars correspond to the numbers of specimens included in the analysis.

Monthly distribution of mean values of RI shows a greater feeding activity during the months of February and March/2006 for *B*. *gauderio* males and females (Fig. 7). According to the analysis of variance (ANOVA) with Tuckey's post-test, male and female RI values differ significantly between the months of sampling (F = 6.92, p<0.05 for males; F = 5.42, p<0.05 for females), March/2006 differing from all the other analyzed months except from February/2006 in males, and from January and February/2006 in females.

**Figure 8:**
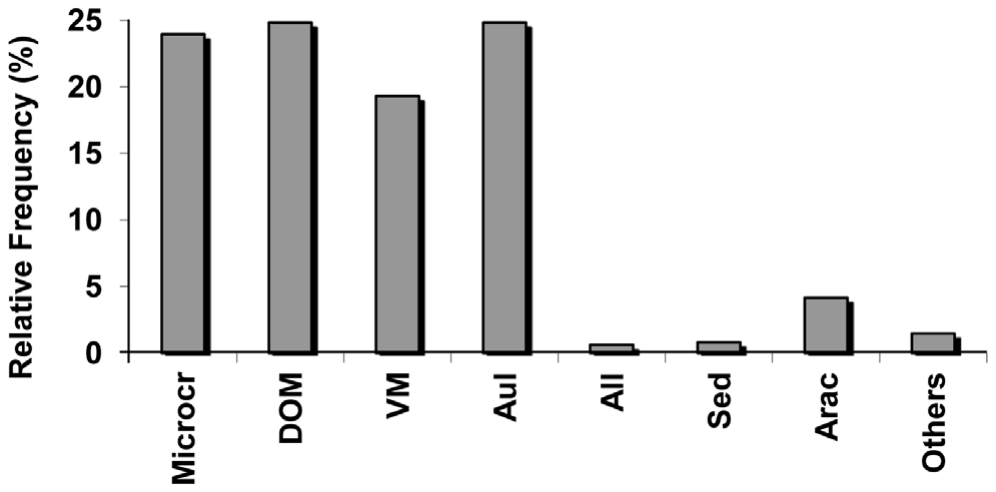
Percent composition of the alimentary categories. Percent composition of the alimentary categories established for *Brachyhypopomus gauderio*. Microc =  microcurstacea; DOM =  digested organic material; VM =  vegetal material; AuI =  autochthonous insects; AlI =  allochthonous insects; Sed =  sediment; Arac =  arachnid; Others =  other items; number of specimens analyzed =  210.

All food items identified through the stomachs analysis are presented in Table 3. There were 23 items identified, the most frequent being microcrustacea (cladocera, copepode, ostracoda), chironomid larvae, insect parts, digested organic material, and vegetal material (Table 3). The percent composition calculation showed the categories AuI and DOM reaching the highest values (24.82%), followed by Microcr (23.96%) and VM (19.31%), whereas the categories Sed and AlI reached the lowest values (0.82% and 0.61% respectively) (Fig. 8). According to the index of alimentary importance, the category DOM is consider main food trough all the sampled months, as well as AuI, that is consider main food during all the months for except April/2004; Microcr is consider additional food during all the year, as well as VM, that is consider additional food trough all the months except for August/2004; while the other categories are consider accidental food during all the sampled period (Table 4). *Brachyhypopomus gauderio* intestinal quotient was estimated as 0.29, with a standard deviation of 0.03 and without significant variation during the months of the studied year.

**Table 3:** Frequency of occurrence.

Item/Month	Apr	May	Jun	Jul	Aug	Sep	Oct	Nov	Dec	Jan	Feb	Mar
Crustacea	100	100	100	93.33	81.25	100	100	83.33	100	100	100	100
Decapoda	0	0	0	0	0	0	0	0	0	0	11.11	0
Microcrustacea	100	100	100	93.33	81.25	100	100	83.33	100	100	100	100
Cladocera	95.24	95	100	80	81.25	100	100	66.67	75	100	100	100
Copepoda	4.76	80	81.25	80	18.75	68.42	80.95	61.11	58.33	30	22.22	30
Ostracoda	4.76	55	50	60	18.75	73.68	52.38	50	83.33	50	100	50
Insecta	100	100	100	100	100	100	100	100	100	100	100	100
Diptera	100	100	100	100	100	100	100	100	100	100	100	100
non-identified-pupa	4.76	5	0	13.33	0	31.58	19.04	16.67	66.67	40	33.33	10
Chironomidae (larvae)	100	100	100	100	100	100	100	100	100	100	100	100
Chironoidae (pupa)	4.76	25	12.5	0	0	68.42	61.9	72.22	41.67	0	0	0
Hymenoptera*	0	0	0	0	0	0	9.52	0	0	0	0	0
Formicidae*	0	0	0	0	0	0	9.52	0	0	0	0	0
Ephemeroptera (naiad)	0	0	12.5	46.67	0	42.1	28.57	11.11	0	0	0	0
Ephemeroptera (larvae)	28.57	65	50	53.33	6.25	42.1	71.43	72.22	58.33	20	6.67	10
Coleoptera (adult)	0	0	0	0	0	0	4.76	0	8.33	0	0	0
Coleoptera (larvae)	47.62	30	6.25	53.33	25	47.37	85.71	100	83.33	70	60	45
Odonata (naiad)	14.28	1	6.25	40	0	15.79	23.81	33.33	33.33	0	0	10
Odonata (larvae)	14.28	30	18.75	26.67	6.25	15.79	33.33	11.11	50	70	40	25
Insect parts	71.43	85	50	80	37.5	89.47	90.48	77.78	91.67	90	80	75
Non-identified-larvae	0	0	0	6.67	6.25	0	4.76	5.55	8.33	0	0	0
Aracnida	23.81	15	12.5	0	12.5	10.53	4.76	11.11	41.67	40	20	25
Acarina	23.08	15	12.5	0	12.5	10.53	4.76	11.11	33.33	40	20	20
Acarina*	0	0	0	0	0	0	0	0	8.33	0	0	5
Sponge spicule	4.76	5	6.25	0	6.25	5.26	14.28	0	0	0	0	10
Mollusc gastropod	0	0	0	0	0	0	0	0	0	0	0	5
Digested organic material	100	100	100	100	100	100	100	100	100	100	100	100
Vegetal material	90.48	80	75	66.67	56.25	94.74	95.24	77.78	75	70	73.33	85
Sediment	0	10	12.5	0	0	15.79	0	0	0	10	0	5
N	21	20	16	15	16	19	21	18	12	14	15	20

Frequency of occurrence (%) of food items identified in stomachs of *Brachyhypopomus gauderio.* *  =  items of allochthonous origin; N =  number of specimens analyzed.

**Table 4:** Alimentary importance index.

Item/Month	Apr	May	Jun	Jul	Aug	Sep	Oct	Nov	Dec	Jan	Feb	Mar
Microcr	0.25	0.25	0.28	0.23	0.22	0.25	0.25	0.21	0.29	0.25	0.25	0.25
DOM	**0.9**	**0.74**	**0.64**	**0.6**	**0.83**	**0.5**	**0.56**	**0.55**	**0.56**	**0.62**	**0.72**	**0.74**
VM	0.24	0.2	0.19	0.17	0.14	0.21	0.24	0.19	0.17	0.17	0.15	0.21
AuI	0.28	**0.31**	**0.47**	**0.54**	**0.31**	**0.49**	**0.49**	**0.72**	**0.5**	**0.35**	**0.38**	**0.36**
AlI	0	0	0	0	0	0	0.03	0	0.04	0	0	0
Sed	0	0.02	0.01	0	0	0.04	0.02	0	0	0	0	0
Arac	0.04	0.03	0.03	0	0.03	0.03	0.02	0.04	0.08	0.1	0.05	0.06
Others	0.01	0.01	0.01	0	0.01	0	0.03	0.01	0	0	0.02	0.04

Alimentary importance index (AII) for the food categories of *Brachyhypopomus gauderio*. Microc =  microcurstacea; DOM =  digested organic material; VM =  vegetal material; AuI =  autochthonous insects; AlI =  allochthonous insects; Sed =  sediment; Arac =  arachnid; Others =  other items. Bold values =  main food category; framed values =  additional food category; simple values =  accidental food category; number of specimens analyzed =  210.

## Discussion

Life histories are shaped by the interaction of extrinsic and intrinsic factors, where the extrinsic factors are ecological impacts on survival and reproduction, and the intrinsic factors are the tradeoffs among life history traits and lineage-specific constraints on the expression of genetic variation [39].The reproductive period established herein for *B*. *gauderio* occurred during the South Hemisphere spring and summer. Positive relation between male and female reproductive season and the photoperiod variation of the study area was recognized. No statistically significant relation between reproduction and the temperature variation was found, even though the reproductive season of the species occurs at the period of the year when the highest water temperature values were registered. This result might be explained by the fact that, at the collect site the temperature does not present a uniform and continual annual variation. These observations are in agreement with the results from many recent studies concerning gymnotiforms from Southern Brazil and Uruguay [10], [11], [13]–[19], where photoperiod and/or temperature trigger reproductive period, and in disagreement with the results showed for gymnotiforms occurring in the tropical weather region of South America [2]–[6], [8], [9], [12], where increasing rainfall precipitation and water level determine reproduction. Many authors compare exogenous factors that determine the annual cycles of fishes under tropical and temperate climatic conditions [38], [40]–[42]. The great majority of the studied neotropical fishes present seasonal reproduction characterized by reproductive periods intercalated with resting periods [42]. The reproductive seasonality in the fishes of temperate environments, where longer rainfall periods are not definite, is mainly related to the temperature, photoperiod, and food availability [43], [44]. However, in tropical environments, the annual temperature and photoperiod variation are quite small, rainfall and habitat availability becoming the factors responsible for the seasonality in the rivers, streams, and lagoons [45]–[47]. Therefore, according to the results obtained for *B*. *gauderio* and the species studied up until now, these ecological patterns may be applied for gymnotiform fishes. Gymnotiform species from different genus and even families, but occurring in the same region, show identical patterns of reproductive period establishment. However, the same genus species, this time occurring in different climatic regions, show divergent reproductive patterns. These findings suggest that environmental conditions influence reproductive period determination in Gymnotiformes more strongly than the degree of relationship between the taxa, being an adaptative condition instead of an inherited character.

According to Winemiller [48], distinct reproductive patterns displayed by the species are adaptations to temporal and spatial variation in the environmental settings, food availability, and predation pressure. The gymnotiform species from the southern boundary of the order distribution, including *B*. *gauderio* according to the results herein obtained, present long reproductive periods [16]–[18] and low values of relative fecundity [16]–[19]. As well as *B*. *gauderio*, these species show high first gonadal maturation sizes [16], [18], [19], which are superior to 40% of the maximum length of the species fitting the concept of late maturation [49], and almost all have parental care behavior described or observed [16, 18, Giora unpublished data for *Brachyhypopomus draco*]. All those quoted life history characters agree with the “K-strategy” as originally proposed by Pianka [50], and with the “equilibrium strategy” as proposed by Winemiller [51]. Both hypotheses are associated with higher juvenile survivorship as a result of greater parental investment in individual progeny. Fractional spawning is also a trait associated with the life history strategies quoted above, and has been registered for *B*. *gauderio* and all the gymnotiform species studied up until now from high [15]–[19], [52] and low latitudes [7], [12], [53], [54], being consider as a general characteristic of the order.

Winemiller [48] analyzed the reproductive traits and population variations of 71 freshwater fishes during one year of sampling in a stream in Venezuela and defined as seasonal strategy the species *Adontosternarchus devenanzii* – Apteronotidae, *Hypopomus* sp. – Hypopomidae, and *Eigenmannia virescens* – Sternopygidae, and as equilibrium strategy the species *Gymnotus carapo* – Gymnotidae. Hydrological and flow regimes have a fundamental role as a key determinant of fish life history composition across a broad biogeographical scale [55]. The occurrence of equilibrium strategist species is associated with decreasing hydrologic variability and increasing stability of environmental flow [55]–[57], while the occurrence of seasonal strategist species is strongly related to environments with high flow seasonality [55], [56]. With this mind and comparing the results obtained for species occurring in environments with stable hydrological regimes in southern Brazil and Uruguay [16]–[19] with species occurring in environments with elevated flow seasonality in Venezuela [48], we can infer that flow regimes have also a great influence in the determination of gymnotiform life history strategy. However, considering the lack of data on life history of gymnotiforms from the northern portion of their distribution – *e*.*g*. relative fecundity, first gonad maturation size, and duration of the reproductive period – the definition of reproductive patterns and strategies for the all order representatives occurring in that region still needs further investigation.

The mechanisms of sexual selection promote the emerging and fixation of dimorphic characters through the choice of males by females and the competition between males or vice-versa [58]. No sexual dimorphism related to the fish total length has been documented; however, *B*. *gauderio* has exhibited a sexually dimorphic shape of the caudal filament distal portion. The majority of the *Brachyhypopomus* species – such as *B. pinnicaudatus, B. beebei, B. brevirostris, B. occidentalis,* and *B. draco* - possesses some kind of broadening, compression, and/or elongation of the mature male caudal filaments [9], [17], [58]–[60]. These morphological modifications of caudal filament may allow males to have greater electrocytes than females, to produce the sexually dimorphic signals [59], and to accrue more matings [9]. It has been reported for a *B. draco* population that males undergo hypertrophy of the distal portion of caudal filament during the reproductive period, after that regressing this structure until the caudal filament resembles those of females and juveniles [17]. The existence of similar caudal filament regression cannot be affirmed for *B*. *gauderio*, since males with hypertrophy of this structure could be found along all months in which large males were sampled. The highest frequency of specimens with broadened filament along the reproductive period months shows that the development of this structure probably occurs concomitantly with male gonadal maturation. However, the presence of males with the highest total lengths exhibiting caudal filaments identical to those of females and juveniles suggests a dominance system among males. Because of that, just dominant males undergo caudal filament hypertrophy. Dominance among males of a same breeding group has been also observed for the species *B. brevirostris* during agnostic behavior observed in captivity [12].

The feeding ecology of a species, is thoroughly linked to its population dynamics, and contributes to the understanding of subjects such as resource partitioning, habitat preference, prey selection, predation, evolution, risk effects, competition and trophic ecology, and energy transfer within and between ecosystems [61]. The values of RI of males and females show greater feeding activity for *B*. *gauderio* just after the reproductive period of the species, suggesting this as the period of energy accumulation, the other months of the year do not present any significant increase or decrease in food consumption. The great amount of digested organic material in the stomachs of *B*. *gauderio* can be attributed to the advanced decomposition state of the ingested food items, in the same way it has been quoted for *B*. *bombilla* [19]. Since gymnotiforms are mostly nocturnal or crepuscular fishes [62] at the time of fish sampling (between 10:00 am and 16:00 pm), food could be already partially digested in the stomach of the specimens, making the item identification impossible.

There are high frequency of occurrence and high values of percent composition and alimentary importance estimated for vegetal material in the diet of *B*. *gauderio*; which might have been debris consumed during the feeding on benthic and macrophyte dwelling invertebrates. Due to the fact that aquatic invertebrates play the main role in the feeding according to all index tested, the species can be herein classified as invertivorous. According to the scale proposed by Fryer and Iles [63] to compare intestine length and trophic category of a species, carnivorous fish present the lowest values of intestine length, followed by omnivorous, and herbivorous, whereas detritivorous present the highest values. Therefore, the low value of intestinal quotient established for *B*. *gauderio* corroborates the definition of an invertivorous feeding.

The studies regarding feeding habits of gymnotiform species – including *B*. *gauderio* investigated here – show similar composition of diets, with fishes feeding mainly on planktonic and benthonic invertebrates [19], [62], [64]–[73] (Table S1), and many of them having chironomid larvae and/or microcrustacea quoted as main items on their diet [19], [62], [64]–[66], [70]–[72] (Table S1).

Among the studied species there are representatives of all the five gymnotiform families (Sternopygidae, Apteronotidae, Hypopomidae, Rhamphichthyidae, Gymnotidae) occurring in southern Brazil, Amazon basin, Orinoco river, Pantanal floodplain in Mato Grosso do Sul state, Corumbá river basin in Goiás state, Paraná river, Bolivia, and Venezuela [19], [64]–[68], [70]–[74], and inhabiting irrigation channels, “igarapés”, lakes, main river channels, floodplains, and creeks [19], [64]–[68], [70]–[74] (Table S1). Therefore, we were lead to conclude that there is no variation on the diet of gymnotiform species related to climatic conditions or even to habitat of occurrence. The family Gymnotidae has in the species *Electrophorus electricus*, an exception of this pattern. The “poraquê”, *E*. *electricus*, is a carnivorous species known to feed on fishes, small aquatic vertebrates, crustaceous, and insect larvae [74]. Species of the genus *Gymnotus* – Gymnotidae family – have been considered predators of aquatic insects and small fishes [22]. However, *Gymnotus carapo* is mainly referred to in the literature as feeding mainly on aquatic insects [65], [66], [71], [72] (Table S1), the same preys being quoted for *Gymntous anguilares* from tributaries of Sinnamari river in Venezuela [66], and for a *Gymnotus* species from the *pantherinus* group in southern Brazil (Vanin unpublished data). Fishes was quoted as an additional item to aquatic insects in the diet of *Gymnotus carapo* from Parana river and its floodplain [73], and as the main item of the diet of *Gymnotus carapo* from Caño Maraca, Venezuela [70]. Consequently, based on the results presented, we can state that the order Gymnotiformes is quite conservative concerning alimentary resources and has very low trophic diversity, especially when compared to other orders such as Siluriformes or Characiformes or even to highly diverse families such as Characidae, this statement contradicting what was expected based on the great variety of head and oral jaw morphology among its genera and species.

## Supporting Information

Table S1 **Feeding habits of gymnotiforms.** Published data concerning the feeding habits of species of the order Gymnotiformes from different localities through the Neotropical region.
(DOC)

## References

[1] Crampton WGR (1998) Efects of anoxia on the distribution, respiratory strategies and electric diversity of gymnotiform fishes. J Fish Biol 53: 307–330.

[2] Hopkins CD (1974a) Electric communication: functions in the social behavior of *Eigenmannia virescens* . Behaviour 50: 270–305.

[3] Hopkins CD (1974b) Electric communication in the reproductive behavior of *Sternopygus macrurus* (Gymnotoidei). Z Tierpsychol 35: 518–535. 4456905 10.1111/j.1439-0310.1974.tb00465.x

[4] Kirschbaum F (1975) Environmental factors control the periodical reproduction of tropical electric fish. Experientia 31: 1159–1160.

[5] Kirschbaum F (1979) Reproduction of the weakly electric fish *Eigenmannia virescens* (Rhamphichtyidae, Teleostei) in captivity. Behav Ecol Sociobiol 4: 331–355.

[6] Kirschbaum F (1984) Reproduction of weakly electric teleosts: just another example of convergent development? Environm Biol Fish 10(1/2): 3–14.

[7] Kirschbaum F (2000) The breeding of tropical freshwater fishes through experimental variation of exogenous parameters. Breedin through simulation of high and low water conditions. Aquageografia 20: 95–105.

[8] Schwassmann HO (1976) Ecology and taxonomic status of different geographic populations of *Gymnorhamphichthys hypostomus*, Ellis (Pisces, Cypriniformes, Gymnotoidei). Biotropica 8: 25–40.

[9] Hagedorn M (1988) Ecology and behavior of a pulse-type electric fish, *Hypopomus occidentalis* (Gymnotiformes, Hypopomidae), in a fresh-water stream in Panama. Copeia 1988(2): 324–335.

[10] Silva A, Quintana L, Galeano M, Errandonea P, Macadar O (1999) Water temperature sensitivity of EOD waveform in *Brachyhypopomus pinnicaudatus* . J Comp Physiol A 185: 187–197 10.1007/s003590050377

[11] Ardanaz JL, Silva A, Macadar O (2001) Temperature sensitivity of the electric organ discharge waveform in *Gymnotus carapo* . J Comp Physiol A 187: 853–864. 10.1007/s00359-001-0256-8 11866184

[12] Kirschbaum F, Schugardt C (2002) Reproductive strategies and developmental aspects in mormyrid and gymnotiform fishes. J Physiol - Paris 96(2002): 557–566. 14692503 10.1016/S0928-4257(03)00011-1

[13] Silva A, Quintana L, Ardanaz JL, Macadar O (2002) Environmental and hormonal influences upon EOD waveform in gymnotiform pulse fish. J Physiol 96: 473–484. 10.1016/S0928-4257(03)00003-2 14692495

[14] Silva A, Quintana L, Galeano M, Errandonea P (2003) Biogeography and breeding in Gymnotiformes from Uruguay. Environm Biol Fish 66: 329–338 10.1023/a:1023986600069

[15] Quintana L, Silva A, Berois N, Macadar O (2004) Temperature induces gonadal maturation and affects electrophysiological sexual maturity indicators in *Brachyhypopomus pinnicaudatus* from a temperate climate. J Exper Biol 207: 1843–1853 10.1242/jeb.00954 15107439

[16] Cognato DP, Fialho CB (2006) Reproductive biology of a population of *Gymnotus* aff. *carapo* (Teleostei: Gymnotidae) from southern Brazil. Neotrop Ichth 4: 339–348.

[17] Schaan AB, Giora J, Fialho CB (2009) Reproductive biology of the Neotropical electric fish *Brachyhypopomus draco* (Teleostei: Hypopomidae) from southern Brazil. Neotrop Ichth 7: 737–744.

[18] Giora J, Fialho CB (2009) Reproductive biology of weakly electric fish *Eigenmannia trilineata* López & Castello, 1966 (TELEOSTEI, Sternopygidae). Brazil Arch Biol Techn 52: 617–628.

[19] Giora J, Tarasconi HM, Fialho CB (2012) Reproduction and feeding habits of the highly seasonal *Brachyhypopomus bombilla* (Gymnotiformes: Hypopomidae) from southern Brazil with evidence for a dormancy period. Environm Biol Fish 94: : 649–662. doi 10.1007/s10641-011-9971-3

[20] Albert JS (2001) Species diversity and phylogenetic systematics of American knifefishes (Gymnotiformes, Teleostei). Miscellan. Publ Mus Zool Univ of Mich 190: 1–129.

[21] Alves-Gomes JA (1997) Informações preliminares sobre a bio-ecologia de peixes elétricos (Ordem Gymnotiformes) em Roraima. In: Barbosa RI, Ferreira EJG, Castellón EG (eds) Homem, Ambiente e Ecologia no Estado de Roraima. INPA, Manaus, pp. 509–555.

[22] Albert JS, Crampton WGR (2005) Diversity and Phylogeny of Neotropical electric fishes (Gymnotiformes). In: Bullock TE, Hopkins CD, Popper AN, Fay FR (eds.) Electroreception. Cornell University Press, Ithaca, pp 360–409.

[23] Crampton WGR (1996) Gymnotiform fish: an important component of Amazonian floodplain fish communities. J Fish Biol 48: 298–301.

[24] Eschmeyer WN, Fong JD (2011) Species of Fishes by family/subfamily. Electronic Database. http://research.calacademy.org/redirect? url = http://researcharchive.calacademy.org/research/Ichthyology/catalog/fishcatmain.asp. Accessed: 2013 Sept 23.

[25] Triques ML, Khamis DK (2003) *Brachyhypopomus jureiae*, a new species of freshwater Neotropical electric fish (Teleostei: Gymnotiformes: Hypopomidae) from a coastal stream of southeastern Brazil. Lundiana 4: 61–64.

[26] Loureiro M, Silva A (2006) A new species of *Brachyhypopomus* (Gymnotiformes: Hypopomidae) from Northeast Uruguay. Copeia 2006: 665–673 10.1643/0045-8511(2006)6665:ANSOBG2.0.CO2

[27] Giora J, Malabarba LR, Crampton WGR (2008) *Brachyhypopomus draco*, a new sexually dimorphic species of Neotropical electric fish from southern South America (Gymnotiformes: Hypopomidae). Neotrop Ichth 6: 159–168.

[28] Giora J, Malabarba LR (2009) *Brachyhypopomus gauderio*, new species, a new example of underestimated species diversity of electric fishes in the southern South America (Gymnotiformes: Hypopomidae). Zootaxa 2093: 60–68.

[29] Crampton WGR, Wells JK, Smyth C, Walz SA (2007) Design and construction of an electric fish finder. Neotrop Ichth 5: 425–428.

[30] Santos EP (1978) Dinâmica de populações aplicada à pesca e piscicultura. Edusp, São Paulo.

[31] Zar JH (1999) Biostatistical analysis. Prentice-Hall, New Jersey.

[32] Adebisi AA (1987) The relationships between fecundities, gonadosomatics indices and egg sizes of some fishes of Ogun River, Nigéria. Arch fuer Hydrobiol 111: 151–156.

[33] Vazzoler AEAM (1996) Biologia da reprodução de peixes teleósteos: teoria e prática. Editora da Universidade, Maringá.

[34] Hyslop EJ (1980) Stomach contents analysis; a review of methods and their application. J Fish Biol 17: 411–429.

[35] Hynes HBN (1950) The food of freshwater sticklebacks (*Gasterosteus aculeatus* and *Pygosteus pungitius*), a review of methods used in studies of the food fishes. J Anim Ecol 19: 36–57.

[36] Granado-Lorencio C, Garcia-Novo F (1986) Feeding habits of the fish community in a eutrophic reservoir in Spain. Ekol Polsk 34: 95–110.

[37] Guillen E, Granado C (1984) Alimentacion de la ictiofauna del embalse de Torrejon (rio Tajo, Caceres). Limn 1: 304–310.

[38] Lowe-McConnell RH (1999) Estudos ecológicos de comunidades de peixes tropicais. Edusp, São Paulo.

[39] Stearns SC (2000) Life history evolution:successes, limitations, and prospects. Naturwissenschaften 87: 476–487. 11151666 10.1007/s001140050763

[40] Schwassmann HO (1971) Biological rhythms. In: Hoar WS, Randall DJ (eds) Fish Fisiology, Vol 6. Academic Press, New York, pp 371–428.

[41] Schwassmann HO (1980) Biological rhythms: their adaotative significance. In: Ali MA (ed) Environmental Physiology of Fishes. Plenum Press, New York, pp 613–629.

[42] Vazzoler AEAM, Menezes NA (1992) Síntese dos conhecimentos sobre o comportamento reprodutivo dos Characiformes da América do Sul (Teleostei, Ostariophysi). Rev Bras Biol 52(4): 627–640.

[43] McKaye KR (1984) Behavioral aspects of cichlids reproductive strategies: patterns of territoriality and brood defense in Central American substratum spawners and African mouth brooders. In: Potts GW, Wooton RJ (eds). Fish reproduction: strategies and tactics. Academic Press, London, pp 245–273.

[44] Payne AI (1986) The ecology of tropical lakes and rivers. John Wiley, New York.

[45] Kramer DL (1978) Reproductive seasonality in the fishes of a tropical stream. Ecology 59: 976–985.

[46] Welcomme RL (1979) Fisheries ecology of floodplain rivers. Longman, Lodon.

[47] Goulding M (1980) The fishes and the forest: explorations in Amazonian natural history. University of California Press, Berkeley.

[48] Winemiller KO (1989) Patterns of variation in life history among South American fishes in seasonal environments. Oecologia 81: 225–241. 28312542 10.1007/BF00379810

[49] Suzuzki HI (1999) Estratégias reprodutivas de peixes relacionadas ao sucesso na colonização em dois reservatórios do rio Iguaçu, PR, Brasil. Dissertation, Universidade Federal de São Carlos.

[50] Pianka ER (1970) On r- and K-selection. Am Nat 100: 592–597.

[51] Winemiller KO (1987) Tests of ecomorphological and community level convergence among neotropical fish assemblages. Dissertation, University of Texas.

[52] Barbieri G, Barbieri MC (1982) Fecundidade e tipo de desova de *Gymnotus carapo* (Linnaeus, 1758), na represa do Lobo, Estado de São Paulo (Pisces, Gymnotidae). Spectr J Bras Ciênc 2: 25–29.

[53] Assunção MIS, Schwassmann HO (1995) Reproduction and larval development of *Electrophorus electricus* on Marajó Island (Pará, Brazil). Ichth Expl Freshw 6: 175–184.

[54] Crampton WGR, Hopkins CD (2005) Nesting and paternal care in the weakly electric fish *Gymnotus* (Gymnotiformes: Gymnotidae) with descriptions of larval and adult electric organ discharges of two species. Copeia 2005: 48–60.

[55] Mims MC, Olden JD (2012) Life history theory predicts fish assemblage response to hydrologic regimes. Ecology 93(1): 35–45. 22486085 10.1890/11-0370.1

[56] Tedesco PA, Hugueny B, Oberdorff T, Durr HH, Merigoux S, et al (2008) River hydrological seasonality influences life history strategies of tropical riverine fishes. Oecologia 156: 691–702. 18368426 10.1007/s00442-008-1021-2

[57] Olden JD, Kennard MJ (2010) Intercontinental comparison of fish life history strategies along a gradient of hydrologic variability. In: Gido KB, Jackson DA (eds) Community Ecology os Stream fishes: concepts, approaches, and techniques. American Fisheries Society, Bethesda, pp 83–107.

[58] Rapp Py-Daniel LH, Cox-Fernandes C (2005) Dimorfismo sexual em Siluriformes e Gymnotiformes (Ostariophysi) da Amazônia. Acta Amazonica 35(1): 97–110.

[59] Hagedorn M, Carr E (1985) Single electrocytes produce a sexually dimorphic signal in South American electric fish. J Comp Physiol 156: 511–523.

[60] Hopkins CD, Comfort NC, Bastian J, Bass AH (1990) Functional analysis of sexual dimorphism in an electric fish, *Hypopomus pinnicaudatus*, Order Gymnotiformes. Brain Behav Evol 35: 350–367. 2245315 10.1159/000115880

[61] Braga RR, Bornatowski H, Vitule JRS (2012) Feeding ecology of fishes: an overview of worldwide publications. Rev Fish Biol Fisheries 22: 915–929 10.1007/s11160-012-9273-7

[62] Mago-Leccia F (1994) Electric fishes of continental waters of America. Clemente Editores, Caracas.

[63] Fryer G, Iles TD (1972) The cichlid fishes of the great lakes of Africa. Oliver & Boyd, Edinburgh.

[64] Giora J, Fialho CB, Dufech APS (2005) Feeding habit of *Eigenmannia trilineata* Lopez & Castello, 1996 (Teleostei: Sternopygidae) of Parque Estadual de Itapuã, RS, Brazil. Neotrop Ichth 3: 291–298.

[65] Soares MGM (1979) Aspectos ecológicos, alimentação e reprodução dos peixes do igarapé do Porto, Aripuanã, MT. Acta Amaz 9: 325–352.

[66] Mérigoux S, Ponton D (1998) Body shape, diet and onto-genetic diet shifts in young fish of the Sinnamary River, French Guiana, South America. J Fish Biol 52: 556–569.

[67] Lundberg JG, Lewis WM Jr, Saunders JF III, Mago-Leccia F (1987) A major food web component in the Orinoco river channel: evidence from planktivorous electric fishes. Science 237: 81–83. 17813624 10.1126/science.237.4810.81

[68] Pouilly M, Lino F, Bretenoux JG, Rosales C (2003) Dietary–morphological relationships in a fish assemblage of the Bolivian Amazonian floodplain. J Fish Biol 62: 1137–1158.

[69] Arrington AD, Winemiller KO, Loftus WF, Akin S (2002) How often do fishes "run on empty"? Ecology 83: 2145–2151.

[70] Winemiller KO, Adite A (1997) Convergent evolution of weakly electric fishes from floodplain habitats in Africa and South America. Environm Biol Fish 49: 175–186.

[71] Luz-Agostinho KDG, Bini LM, Fugi R, Agostinho AA, Júlio Jr HF (2006) Food spectrum and trophic structure of the ichthyofauna of Corumbá reservoir, Paraná river Basin, Brazil. Neotrop Ichth 4(1): 61–68.

[72] Resende EK, Pereira RAC, Sório VF, Galvão EM (2006) Biologia da tuvira, *Gymnotus* cf. *carapo* (Pisces, Gymnotidae) no Baixo Rio Negro, Pantanal, Mato Grosso do Sul, Brasil. Bol Pesq Desenvolv Embrapa 2006: 1–42.

[73] Penczak T, Agostinho AA, Hahn N (2000) Na ordination technique for fis diet comparison. Bras Achiv Biol Techn 43(1): 101–101.

[74] Crampton WGR, Ribeiro AC (2013) Gymnotidae. In: Queiroz LJ, Torrente-Vilara G, Ohara WM, Pires THS, Zuanon J, Doria CRC, (eds) Peixes do Rio Madeira Volume III, Santo Antônio Energia, São Paulo pp 207–217.

