# Supplementary material for: Reproduction and Feeding of the Electric Fish Brachyhypopomus gauderio (Gymnotiformes: Hypopomidae) and the Discussion of a Life History Pattern for Gymnotiforms from High Latitudes
Source: PLoS One. 2014 Sep 10;9(9):e106515. doi: 10.1371/journal.pone.0106515 (PMC4160160; doi:10.1371/journal.pone.0106515)
Supplement: Table S1 — Feeding habits of gymnotiforms. Published data concerning the feeding habits of species of the order Gymnotiformes from different localities through the Neotropical region. (DOC) [file pone.0106515.s001.doc]

**Table S1. Feeding habits of gymnotiforms**

**Table S1.** Published data concerning the feeding habits of species of the order Gymnotiformes from different localities through the Neotropical region.

| Family/*Species* | Feeding | Habitat | Reference |
| --- | --- | --- | --- |
| Sternopygidae |  |  |  |
| *Eigenmannia virescens* | autochthonous insects (mainly larvae) | “igarapé” in Amazon basin in Mato Grosso state | 65 |
| *Eigenmannia virescens* | aquatic invertebrates | lakes of the floodplain of Mamoré river, Bolivia | 68 |
| *Eigenmannia virescens* | zooplankton and other microcrustacea, and aquatic Diptera larvae | Caño Maraca, Venezuela | 70 |
| *Eigenmannia trilineata* | microcrustacea, autochthonous insects (mainly chironomidae larvae) | irrigation channel aside to a lagoon at Laguna dos Patos basin, Rio Grande do Sul | 64 |
| *Eigenmannia trilineata* | aquatic insects and detritus | Parana river and its floodplain | 73 |
| *Eigenmannia* sp. | aqutic insects and microcrustaceans | Corumbá river basin, Goiás State | 71 |
| *Rhabdolichops zareti* | zooplankton and dipteran larvae (almost exclusively chironomidae larvae) | main channel of the Orinoco river | 67 |
| *Sternopygus macrurus* | insect larvae | tributaries of Sinnamari river in Venezuela | 66 |
| *Sternopygus macrurus* | aquatic insects | Caño Maraca, Venezuela | 70 |
| Apteronotidae |  |  |  |
| *Adontosternarchus sachsi* | aquatic invertebrates | lakes of the floodplain of Mamoré river, Bolivia | 68 |
| *Adontosternachus devananzii* | aquatic Diptera larvae | Caño Maraca, Venezuela | 70 |
| *Apteronotus albifrons* | terrestrial arthropods and aquatic Diptera larvae | Caño Maraca, Venezuela | 70 |
| Hypopomidae |  |  |  |
| *Brachyhypopomus beebei* | insect larvae | tributaries of Sinnamari river in Venezuela | 66 |
| *Brachyhypopomus bombilla* | autochthonous insects (mainly chironomidae larvae) | creek at Uruguay basin, Rio Grande do Sul | 19 |
| *Brachyhypopomus gauderio* | autochthonous insects (mainly chironomidae larvae) | creek at Laguna dos Patos basin, Rio Grande do Sul |  |
| *Brachyhypopomus occidentalis* | aquatic Diptera larvae, and zooplankton and other microcrustacea | Caño Maraca, Venezuela | 70 |
| *Hypopomus artedi* | insect larvae | tributaries of Sinnamari river in Venezuela | 66 |
| Rhamphichthyidae |  |  |  |
| *Rhamphichthys marmoratus* | aquatic insects, and zooplankton and other microcrustacea | Caño Maraca, Venezuela | 70 |
| *Rhamphichthys rostratus* | aquatic insects | Parana river and its floodplain | 73 |
| Gymnotidae |  |  |  |
| *Gymnotus carapo* | mainly insects with contribution of fishes | Parana river and its floodplain | 73 |
| *Gymnotus carapo* | autochthonous insects (mainly larvae) | “igarapé” in Amazon basin in Mato Grosso state | 65 |
| *Gymnotus carapo* | insect larvae | tributaries of Sinnamari river in Venezuela | 66 |
| *Gymnotus carapo* | aquatic insects | Corumbá river basin, Goiás State | 71 |
| *Gymnotus carapo* | mainly fishes | Caño Maraca, Venezuela | 70 |
| *Gymnotus* cf. *carapo* | aqutic insects and microcrustaceans | Rio Negro floodplain, Pantanal, Mato Grosso do Sul | 72 |
| *Gymnotus anguilaris* | insect larvae | tributaries of Sinnamari river in Venezuela | 66 |
| *Electrophorus electricus* | fishes, small aquatic vertebrates, crustaceous, and insect larvae |  | 74 |
